# Effects of 14 days of head‐down bed rest with or without exercise, and subsequent recovery on bone turnover, density and structure in older adults

**DOI:** 10.1113/EP093753

**Published:** 2026-06-17

**Authors:** Guy Hajj‐Boutros, Andréa Faust, Carmelo Mastrandrea, Vita Sonjak, Dounia Rouabhia, Gustavo Duque, Richard Kremer, Stéphanie Chevalier, Julie Lacombe, Mathieu Ferron, Tyler A. Churchward‐Venne, José A. Morais

**Affiliations:** ^1^ Research Institute of the McGill University Health Centre Montréal Quebec Canada; ^2^ Schlegel‐University of Waterloo Research Institute for Aging Waterloo Ontario Canada; ^3^ Division of Geriatric Medicine, Faculty of Medicine Laval University Quebec Quebec Canada; ^4^ Department of Kinesiology and Physical Education McGill University Montréal Quebec Canada; ^5^ School of Human Nutrition McGill University Montréal Quebec Canada; ^6^ Molecular Physiology Research Unit Institut de Recherches Cliniques de Montréal Montréal Quebec Canada; ^7^ Department of Medicine Université de Montréal Montréal Québec Canada; ^8^ Division of Geriatric Medicine, Faculty of Medicine McGill University Montréal Quebec Canada

**Keywords:** bed rest, bone, exercise countermeasure, geriatric, space health

## Abstract

Bed rest accelerates bone loss and may exacerbate skeletal fragility. This study examined the effects of 14 days of head‐down tilt bed rest (HDBR) with or without exercise, and subsequent recovery, on bone turnover, density and structure in older adults. Twenty‐two healthy older adults (55–65 years) completed the HDBR protocol. Participants were randomized to a control group that received passive physiotherapy (CON, *n* = 11) or a group that performed daily exercise (EX, *n* = 11). Serum biomarkers of bone formation (procollagen type 1 N‐terminal propeptide (P1NP) and bone‐specific alkaline phosphatase (BSAP)), resorption (N‐terminal cross‐linked telopeptide of type I collagen (NTX) and C‐terminal cross‐linked telopeptide of type I collagen (CTX)), and osteocalcin were measured at baseline (BDC4), day‐9 (HDT9), and immediately (R1), 4 weeks (4W), and 4 months (4M) post‐HDBR. Bone mineral density (BMD) was assessed via dual‐energy X‐ray absorptiometry (DXA) at BDC4, R1, 4W and 4M. Femoral bone structure was measured via peripheral quantitative computed tomography (pQCT) at BDC4 and R1. CTX and NTX increased at R1 vs. BDC4 (time: *P *< 0.001), while P1NP and BSAP increased at 4W and 4M (time: *P *< 0.001). No DXA‐derived BMD changes occurred. pQCT revealed reduced femoral trabecular volumetric BMD at 4% (EX: 342.3 ± 7.8 to 337.9 ± 8.0 mg/cm^3^; CON: 329.3 ± 8.1 to 326.4 ± 8.4 mg/cm^3^; time: *P* = 0.05) and cortical volumetric BMD at 25% (EX: 1089.5 ± 6.7 to 1087.5 ± 7.1 mg/cm^3^; CON: 1102.7 ± 6.7 to 1095.7 ± 7.1 mg/cm^3^; time: *P* = 0.05). However, changes were within the precision error of pQCT measurements. Fourteen days of HDBR, with or without exercise, increased biomarkers of bone resorption but did not alter BMD or bone structure.

## Abstract (highlights)

- **What is the central question of this study?**
  What are the effects of 14 days of head‐down tilt bed rest (HDBR), with or without an exercise countermeasure, and subsequent recovery on serum biomarkers of bone turnover, bone density and bone structure in healthy older adults?
- **What is the main finding and its importance?**
  HDBR increased serum concentration of biomarkers of bone resorption, while recovery increased biomarkers of bone formation. There was no change in bone mineral density at the regions examined. HDBR resulted in small changes in femoral bone structure that fell within established precision error of peripheral quantitative computed tomography measurements.

## 1 INTRODUCTION

Extended periods of physical inactivity, such as those experienced during bed rest, lead to rapid and pronounced declines in physiological function (Sibonga et al., 2007). Head‐down tilt bed rest (HDBR) at 6° is an established terrestrial model that simulates the unloading effects of microgravity, enabling controlled investigation of musculoskeletal adaptations and evaluation of potential countermeasures (Hargens & Vico, 2016). Prolonged disuse of the musculoskeletal system during HDBR results in substantial bone loss, particularly in the lower extremities (Leblanc et al., 1990; Rittweger et al., 2009), with the most pronounced reductions occurring in the cortical bone of the epiphyseal regions (Rittweger et al., 2009). At the level of bone metabolism, skeletal unloading during bed rest appears to shift remodelling toward net bone loss by creating an imbalance between bone resorption and bone formation. Evidence for this imbalance includes a rapid rise in biomarkers of collagen degradation, such as N‐terminal cross‐linked telopeptide of type I collagen (NTX), C‐terminal cross‐linked telopeptide of type I collagen (CTX) and helical peptide (H‐peptide) (LeBlanc et al., 1995; Morgan et al., 2012), alongside no or only modest changes in biomarkers of bone formation including bone‐specific alkaline phosphatase (BSAP) and osteocalcin (OC) (Baecker et al., 2003; Buehlmeier et al., 2017; Kim et al., 2003; Lueken et al., 1993). However, most short‐duration HDBR bone biomarker data come from studies in younger adults. It remains unclear whether older adults show the same time course, magnitude and recovery profile of biomarkers of bone turnover, and whether an exercise countermeasure can modify the response.

Clinically, dual‐energy X‐ray absorptiometry (DXA) has been widely used since the early 2000s to assess bone mineral density (BMD) and fracture risk (Blake & Fogelman, 2009). However, total body DXA is not particularly sensitive to short‐term changes in BMD among young adults, even under extreme conditions such as bed rest (Pouilles et al., 1991). Several studies have demonstrated that detectable reductions in BMD typically require extended periods of mechanical unloading, generally exceeding 60 days, before becoming apparent via DXA (Cavanagh et al., 2016; Smith et al., 2008). Notably, no studies to date have examined site‐specific BMD changes (e.g., femoral neck, lumbar spine) in older adults undergoing 14 days of HDBR.

Beyond biomarkers of bone turnover and DXA‐based assessments of BMD, peripheral quantitative computed tomography (pQCT) has provided valuable insights into structural changes in both trabecular and cortical bone compartments following periods of bed rest. Notably, pQCT or high resolution pQCT imaging has revealed compartment‐specific bone loss after 35 (Rittweger et al., 2009), 60 (Armbrecht et al., 2010, 2011) and 89 days of bed rest (Rittweger et al., 2005). Collectively, these findings highlight pQCT's ability to quantify compartment‐specific changes in bone structure and volumetric density, offering insights into bone strength that are not captured by DXA or circulating biomarkers of bone turnover.

Exercise interventions are widely proposed as effective countermeasures against bed rest‐induced bone loss (Hedge et al., 2022; Konda et al., 2019). For example, Rittweger et al. (2005) reported that physical activity during 90 days of HDBR in young males helped maintain biomarkers of bone formation and mitigate cortical bone loss. Additionally, Kramer et al. (2017) reported that high‐intensity jump training performed 5–6 time per week prevented the decline in bone mineral content and density of the tibia during 60 days of HDBR in young males. Despite these promising findings in younger adults, the skeletal response to unloading during HDBR, and the efficacy of exercise countermeasures in older adults remains under‐investigated. Ageing is associated with reduced baseline bone mass, diminished mechanical loading, and hormonal changes, all of which may blunt the osteogenic response to physical activity. Thus, defining skeletal responses to acute unloading in older adults, and testing whether an exercise countermeasure can mitigate these changes, is essential to (i) improve the physiological relevance of HDBR as a spaceflight analogue for ageing crewmembers and (ii) advance our understanding of disuse‐related bone loss in older, earth‐bound populations as periods of disuse (e.g., bed rest or immobilization due to illness, injury or surgery) occur more frequently in older adults.

The present study investigated the effects of HDBR, with and without an exercise countermeasure and subsequent recovery, on serum biomarkers of bone turnover, DXA‐derived BMD, and pQCT‐derived bone structure in an older adult population. It was hypothesized that HDBR would result in elevations in serum biomarkers of bone resorption and reductions in bone mass, and that these effects would be attenuated in participants who performed a multimodal exercise countermeasure during bed rest.

## 2 METHODS

### 2.1 Ethical approval

All participants provided written informed consent prior to participating in the study. The study conformed to the *Declaration of Helsinki* and was approved by the research ethics committee of the McGill University Health Center (MP‐37‐2021‐7170). The trial was registered at ClinicalTrials.gov (identifier: NCT04964999).

### 2.2 Overview of the study design

The present study was a two‐arm randomized controlled trial, in which neither participants nor assessors were blinded. Participants resided for a total of 26 days at the McConnell Centre for Innovative Medicine (CIM) at the Research Institute of the McGill University Health Centre (Montreal, QC), including 5 days of baseline assessments, 14 days of HDBR, and 7 days of recovery. Follow‐up assessments were also conducted at 4 weeks (4W) and 4 months (4M) post‐intervention. Details of the overall study design, including the full set of measurements, data collection procedures, facility set‐up, intervention protocol, recovery phase and follow‐up procedures, have been described previously (Hajj‐Boutros et al., 2024).

### 2.3 Participants’ information

A total of 219 volunteers expressed interest in participating. Of these, 80 were deemed eligible, but 56 declined. In total, 24 participants were enrolled in the study, and 22 participants completed the 14‐day HDBR protocol. One participant withdrew on day 3 of HDBR due to convenience‐related issues, including difficulty maintaining the head‐down tilt position, trouble with bowel movements and the requirement for assistance with basic needs. Another participant was excluded by the investigators due to the group being full. An additional two participants who completed the HDBR protocol were excluded after experiencing an atrial fibrillation event on day 3 of recovery, which was unrelated to the study. Since these two participants completed pre‐ and post‐HDBR testing, their data were retained. In total, 22 participants completed pre‐ and post‐HDBR testing (11 females and 11 males), and 20 participants completed the study in full (11 females and 9 males), including follow‐up assessments at 4W and 4M of recovery after HDBR. Details on participant flow through the study have been reported previously (Hajj‐Boutros et al., 2023).

### 2.4 Exercise intervention

The exercise countermeasure employed in this study has been described in detail previously (Hajj‐Boutros et al., 2024). Briefly, the exercise intervention consisted of three daily sessions that combined cycling‐based aerobic exercise and resistance training. Across the 14‐day HDBR period, participants completed a rotating schedule of (1) high‐intensity interval training (HIIT) cycling, (2) continuous cycling sessions prescribed for 15 or 30 min (as scheduled), and (3) progressive aerobic cycling sessions with workload advanced over time. Resistance exercise sessions were interspersed throughout the week and alternated between emphasis on the upper‐body and lower‐body, ensuring both regions were trained repeatedly across each week. Overall, the schedule was structured to provide approximately 60–75 min of total exercise per day, distributed over three sessions. All exercises were performed in either the 6° head‐down tilt or horizontal position using available equipment, according to the predefined day‐by‐day schedule. Exercise intensity was individually adjusted based on each participant's performance and tolerance. In contrast, the non‐exercising control group received physiotherapy in the 6° head‐down tilt position. This consisted of passive range‐of‐motion movements, including knee, ankle, shoulder and neck mobility. These sessions were administered by a physiotherapist and were performed on a daily basis, similar to the exercise group; however, they were not divided into three separate sessions per day.

### 2.5 Bone turnover biomarkers

Serum concentrations of bone turnover biomarkers were measured in samples collected at baseline prior to HDBR (BDC4), on day 9 of HDBR (HDT9), immediately after HDBR (R1), and at 4 weeks (4W), and 4 months (4M) recovery post‐HDBR. Specifically, procollagen type 1 N‐terminal propeptide (P1NP), bone‐specific alkaline phosphatase (BSAP), N‐terminal telopeptide of type I collagen (NTX), C‐terminal telopeptide of type I collagen (CTX), and both intact and uncarboxylated osteocalcin, were measured at the Research Institute of the McGill University Health Centre or at the Montreal Clinical Research Institute using commercially available immunoassay kits (details provided below). All assays were performed according to the manufacturers’ instructions by trained personnel blinded to group allocation.

For P1NP, a radioimmunoassay kit (Orion Diagnostica, Espoo, Finland) was used. Due to the use of iodine‐125 (^125^I) as a tracer, the assay was conducted under appropriate radioactive safety protocols in a laboratory with a valid radioactivity permit. Given the short half‐life of ^125^I and the limited stability of the kit, P1NP assays were scheduled in advance and completed promptly upon kit delivery, which followed a monthly distribution schedule. Serum samples were aliquoted prior to analysis to avoid freeze–thaw cycles, which could compromise peptide stability. A volume of 120 µL of serum was used per sample, with all measurements performed in duplicate to ensure accuracy.

Serum concentrations of BSAP, NTX, CTX, and both intact and uncarboxylated osteocalcin were measured using enzyme‐linked immunosorbent assay (ELISA) kits. BSAP was quantified using the MicroVue™ BAP assay (QuidelOrtho Corporation, San Diego, CA, USA), while NTX and CTX were measured using the Osteomark® NTx Serum assay (QuidelOrtho Corporation) and the Serum CrossLaps® ELISA (Immunodiagnostic Systems (IDS), Boldon, UK), respectively. Intact osteocalcin was assessed using the Intact Osteocalcin ELISA (Alpco, Salem, NH, USA; cat. no. 38‐OSTHU‐E01), and uncarboxylated osteocalcin was measured with the LEGEND MAX™ Human Uncarboxylated Osteocalcin ELISA kit (BioLegend, San Diego, CA, USA; cat. no. 446707). All ELISA kits were stored under appropriate conditions and used within their designated shelf lives to ensure assay integrity. Serum samples were aliquoted into single‐use vials to prevent degradation associated with repeated freeze–thaw cycles. All assays were performed in duplicate using the recommended serum volume, following the respective manufacturers’ protocols.

### 2.6 Dual energy X‐ray absorptiometry (DXA)

Areal BMD (g/cm^2^) was assessed using DXA with a Lunar Prodigy scanner (GE Medical Systems, Madison, WI, USA, encore 2002 software, version 10.50.086) at the following regions of interest: total body, total hip, left femoral neck, left femoral trochanters and lumbar spine (L1–L4). For lumbar spine scans, participants lay in the supine position with a block‐shaped cushion placed under the legs to reduce lumbar lordosis and improve image clarity. The positioning laser was aligned approximately 5 cm below the umbilicus to ensure inclusion of L5, portions of the iliac crest, and T12 with visible rib structures. For proximal femur scans, the participant's feet were secured using the DualFemur™ positioner to maintain consistent internal femoral rotation. The laser was positioned approximately 4 cm below the greater trochanter or 1 cm below the pubic symphysis at the mid‐thigh, ensuring accurate visualization of the femoral neck and adjacent bony landmarks. The coefficient of variation (CV%) for BMD measurements demonstrated high precision across all regions: 0.67% for total body, 1.05% for the total hip, 1.39% for the left femoral neck, 1.9% for left femoral trochanters and 0.68% for the lumbar spine (L1–L4).

### 2.7 Peripheral quantitative computed tomography

**FIGURE 1:**
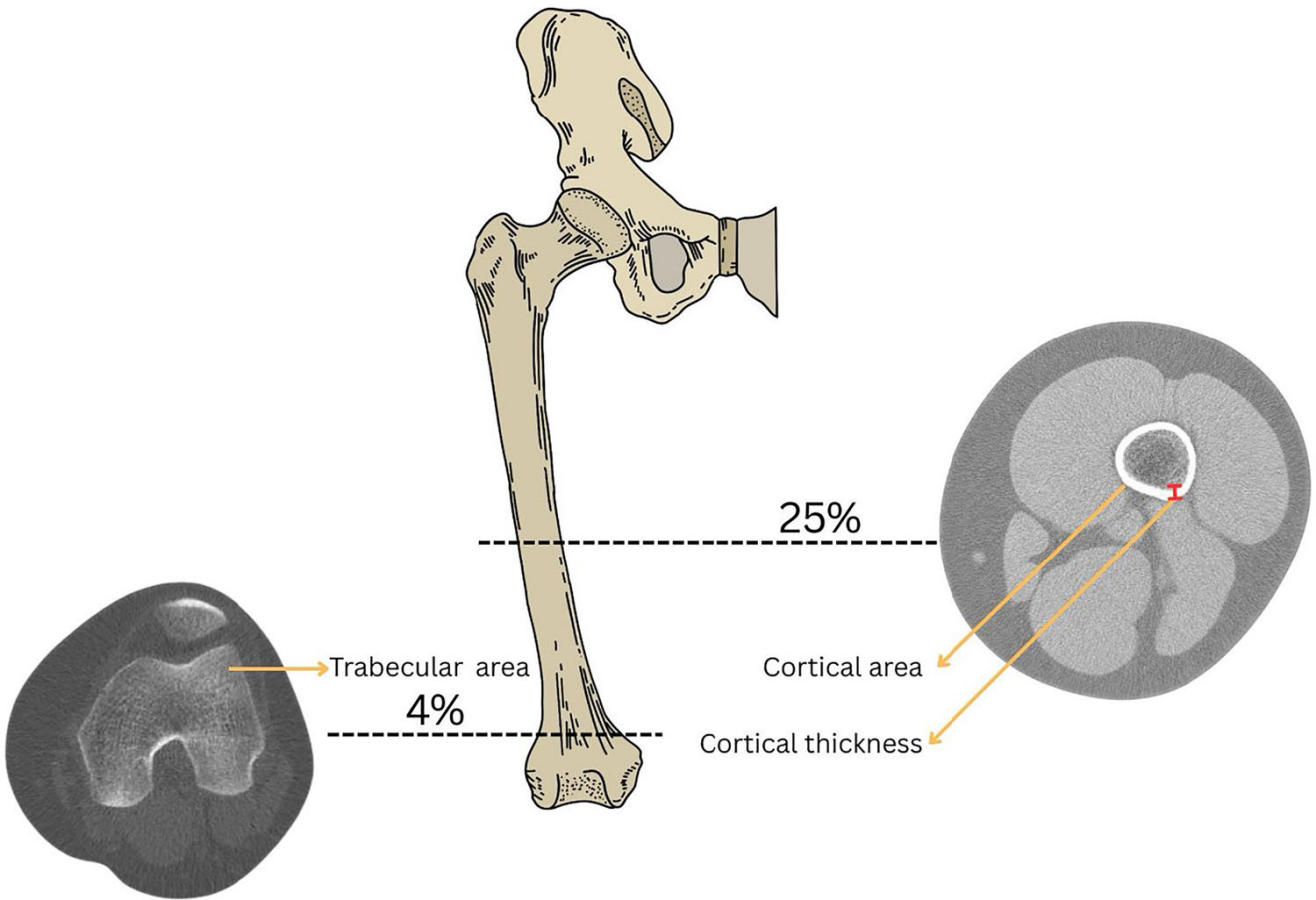
Representative peripheral quantitative computed tomography image showing the femur 4% and 25% site.

pQCT was used to assess volumetric bone mineral density (vBMD) and bone structure at the femur using an XCT 3000 scanner (Stratec Medizintechnik GmbH, Pforzheim, Germany; software version 6.00). All scans were performed by a single trained technician, with daily quality assurance scans to ensure consistent calibration and measurement reliability. Participants were seated and instructed to remain still and breathe normally during scanning. For femoral scans, the dominant leg was measured from the greater trochanter to the lateral epicondyle to determine femur length. A scout view was used to identify the tibial plateau, which served as the anatomical reference point for scan positioning. Once the tibial plateau was localized and the reference line was placed, the scan positions were automatically calculated by the pQCT system to ensure consistency across repeated scans. Standardized thresholds were applied across all analyses, and scans were reviewed for positioning and motion artifacts, with rescans performed when scan quality or positioning was not acceptable. Scans were performed at the 4% (distal epiphysis) and 25% (mid‐diaphysis) sites. At the 4% site, total vBMD, total bone area, and trabecular area were measured using an area threshold of 220 mg/cm^3^ and a BMD threshold of 710 mg/cm^3^. At the 25% site, cortical vBMD, cortical thickness, periosteal and endosteal circumferences, and Stress–Strain Index (SSI) were derived using an area threshold of 280 mg/cm^3^ and the same BMD threshold. In vivo measurement precision was high, with coefficient of variation (CV%) values ranging from 0.70% to 1.20% at the 4% femoral site, and 0.62% to 1.10% at the 25% femoral site, indicating excellent precision across all regions. A representative pQCT image showing the femur 4% and 25% site is shown in Figure 1.

### 2.8 Statistical analysis

Data from twenty‐two participants were included in the analyses (*n* = 11 per group). Participants’ characteristics were summarized via descriptive statistics (mean ± SD). Serum biomarkers of bone turnover were analysed via a mixed effects model with time (BDC4, HDT9, R1, 4W and 4M), group (EX vs. CON) and time × group as fixed effects, and subject as a random effect due to the presence of a limited number of missing data points (25 out of 660 data points across all 6 analytes or 3.8%). Similarly, DXA outcomes were analysed via a mixed effects model with time (BDC4, R1, 4W and 4M), group (EX vs. CON) and time × group as fixed effects, and subject as a random effect due to the presence of a limited number of missing data points (20 out of 440 data points across all 5 outcomes or 4.5%). For pQCT outcomes, a two‐factor repeated measures ANOVA was used to assess the effects of time (BDC4 vs. R1), group (EX vs. CON), and time × group as no missing data were present. If a significant main effect for time or time × group interaction effect was observed, pairwise comparisons with Bonferroni correction were applied when appropriate. Normality of model residuals and the presence of influential observations were checked via visual analysis of QQ plots, while homogeneity of variance was evaluated using visual inspection of residuals versus fitted values plots. Data in text and tables are presented as means ± standard deviation (SD). For all tests, *P* ≤ 0.05 was considered statistically significant. All statistical analyses were performed using GraphPad Prism (v.8.4.3; GraphPad Software, San Diego, CA, USA) and IBM SPSS Statistics software v29 for Mac (IBM Corp., Armonk, NY, USA).

## 3 RESULTS

**FIGURE 2:**
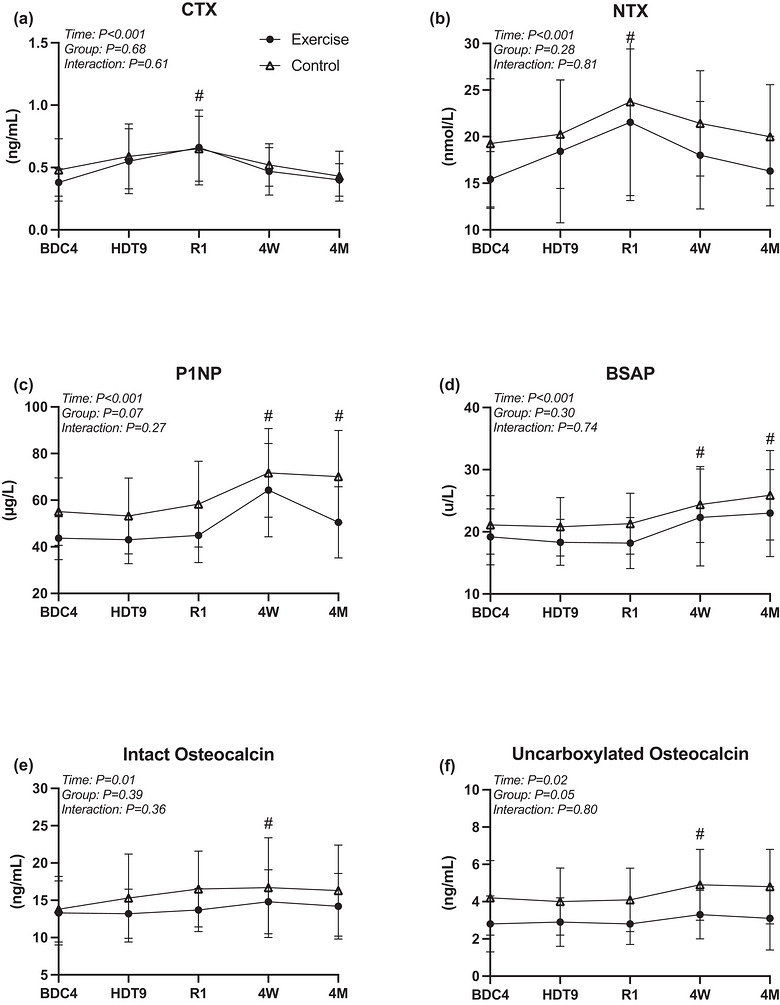
Blood serum biomarkers of bone formation and resorption. #Significant difference from baseline for that time point. 4M, month 4 post‐intervention; 4W, week 4 post‐intervention; BDC4, baseline data collection day 4; HDT9, head down tilt day 9; R1, recovery day 1.

Participants’ baseline characteristics are presented in Table 1. Serum biomarkers of bone resorption including CTX and NTX showed a significant main effect of time (both *P *< 0.001). *Post hoc* pairwise comparisons using Bonferroni correction showed that CTX was only increased immediately following the HDBR intervention compared to baseline (CON–BDC4: 0.48 ± 0.29 ng/mL; R1: 0.64 ± 0.25 ng/mL; EX–BDC4: 0.37 ± 0.1 ng/mL; R1: 0.65 ± 0.3 ng/mL; adjusted *P* = 0.02). Similarly, *post hoc* pairwise comparisons using Bonferroni correction showed that NTX was only increased immediately following the HDBR intervention compared to baseline (CON–BDC4: 19.2 ± 6.9 nmol/L; R1: 23.7 ± 10.5 nmol/L; EX–BDC4: 15.2 ± 2.9 nmol/L; R1: 21.5 ± 7.8 nmol/L; adjusted *P* = 0.01) (Figure 2a, b). There was no evidence of a group × time interaction for CTX or NTX, suggesting that this pattern did not differ between groups. Serum biomarkers of bone formation, including P1NP (Figure 2c) and BSAP (Figure 2d) showed a significant main effect of time (both *P *< 0.001). *Post hoc* pairwise comparisons using Bonferroni correction showed that P1NP was increased at 4W compared to baseline (CON–BDC4: 55.1 ± 14.5 µg/L; 4W: 71.7 ± 19.0 µg/L; EX–BDC4: 43.7 ± 9.2 µg/L; 4W: 64.3 ± 20.0 µg/L; adjusted *P* = 0.001) and 4M compared to baseline (CON–BDC4: 55.1 ± 14.5 µg/L; 4M: 71.1 ± 19.8 µg/L; EX–BDC4: 43.7 ± 9.2 µg/L; 4M: 50.5 ± 15.3 µg/L; adjusted *P* = 0.020). Similarly, *post hoc* pairwise comparisons using Bonferroni correction showed that BSAP was increased at 4W compared to baseline (CON–BDC4: 21.1 ± 4.7 u/L; 4W: 24.4 ± 6.1 u/L; EX–BDC4: 19.2 ± 4.5 u/L; 4W: 22.3 ± 7.8 u/L; adjusted *P* = 0.010) and 4M compared to baseline (CON–BDC4: 21.1 ± 4.7 u/L; 4M: 25.9 ± 7.2 u/L; EX–BDC4: 19.2 ± 4.5 u/L; 4M: 23.0 ± 7.0 u/L; adjusted *P* = 0.001). There was no evidence of a group × time interaction for P1NP or BSAP, suggesting that this pattern did not differ between groups. Intact osteocalcin (Figure 2e) and uncarboxylated osteocalcin (Figure 2f) showed a significant main effect of time (*P* = 0.01 and *P* = 0.02, respectively). *Post hoc* pairwise comparisons using Bonferroni correction showed that intact osteocalcin was increased at 4W following the HDBR intervention compared to baseline (CON–BDC4: 13.8 ± 4.4 ng/mL; 4W: 16.7 ± 6.7 ng/mL; EX–BDC4: 13.3 ± 4.3 ng/mL; 4W: 14.8 ± 4.3 ng/mL; adjusted *P* = 0.005). Similarly, *post hoc* pairwise comparisons using Bonferroni correction showed that uncarboxylated osteocalcin was increased at 4W following the HDBR intervention compared to baseline (CON–BDC4: 4.2 ± 2.0 ng/mL; 4W: 4.9 ± 1.9 ng/mL; EX–BDC4: 2.8 ± 1.5 ng/mL; 4W: 3.3 ± 1.3 ng/mL; adjusted *P* = 0.030). There was no evidence of a group × time interaction for intact or uncarboxylated osteocalcin, suggesting that this pattern did not differ between groups.

**TABLE 1:** Participants characteristics at baseline

	Control	Exercise
*n*	11	11
Sex	F (5), M (6)	F (6), M (5)
Age (years)	58.4 ± 3.9	58.4 ± 3.4
Height (cm)	166.7 ± 10.7	167.1 ± 8.4
Weight (kg)	67.5 ± 14.9	72.4 ± 13.3
BMI (kg/m^2^)	24.0 ± 2.8	25.7 ± 2.9
Systolic blood pressure (mmHg)	123 ± 16	128 ± 9
Diastolic blood pressure (mmHg)	76 ± 12	77 ± 5
Total lean mass (kg)	45.9 ± 10.7	47.8 ± 11.7
Total fat mass (kg)	19.3 ± 5.5	21.9 ± 54.0
Lower limb muscle strength (N)	124.0 ± 48.3	130.1 ± 41.7
${\dot{V}}_{O_{2}\mathrm{peak}}$ (ml/kg/min)	30.2 ± 6.1	32.0 ± 6.9
Glucose (mmol/l)	4.7 ± 0.4	4.8 ± 0.2

*Note*: Values presented as mean ± SD. Abbreviations: BMI, body mass index.

Data on total body, total hip, femoral neck, femoral trochanters, and lumbar spine (L1–L4) bone mineral density (g/cm^2^) measured via DXA are shown in Table 2. For all regions examined, there was no effect of Time (all *P *> 0.05), or group × time interaction (all *P *> 0.05). This suggests that bone mineral density (g/cm^2^) did not change in response to HDBR at R1 or subsequent recovery at 4W and 4M post‐intervention compared to baseline (BDC4), and that this pattern was similar between CON and EX groups.

**TABLE 2:** Total body, total hip, femoral neck, femoral trochanters, and lumbar spine bone mineral density measured with DXA scan.

	Control				Exercise				*P*		
Variables	BDC4	R1	4W	4M	BDC4	R1	4W	4M	Time	Group	Time × Group
Total BMD (g/cm^2^)	1.19 ± 0.07	1.19 ± 0.07	1.18 ± 0.08	1.18 ± 0.09	1.24 ± 0.1	1.24 ± 0.1	1.25 ± 0.14	1.24 ± 0.13	0.18	0.46	0.28
Total hip BMD (g/cm^2^)	0.88 ± 0.02	0.86 ± 0.03	0.87 ± 0.02	0.87 ± 0.03	1.02 ± 0.02	1.02 ± 0.03	1.03 ± 0.11	1.02 ± 0.10	0.053	**<0.001**	0.053
Femoral neck BMD (g/cm^2^)	0.84 ± 0.03	0.81 ± 0.02	0.81 ± 0.08	0.83 ± 0.08	0.95 ± 0.03	0.93 ± 0.02	0.92 ± 0.03	0.93 ± 0.03	0.13	**0.02**	0.22
Trochanter BMD (g/cm^2^)	0.75 ± 0.03	0.74 ± 0.03	0.73 ± 0.09	0.73 ± 0.09	0.87 ± 0.03	0.87 ± 0.03	0.88 ± 0.02	0.87 ± 0.03	0.08	**0.01**	0.09
L1–L4 BMD (g/cm^2^)	1.08 ± 0.03	1.08 ± 0.03	1.07 ± 0.04	1.08 ± 0.09	1.17 ± 0.03	1.15 ± 0.03	1.15 ± 0.07	1.15 ± 0.05	0.12	0.11	0.24

*Note*: Values presented as means ± SD. *P*‐values shown in bold indicate statistical significance. Abbreviations: BMD, bone mineral density; DXA, dual‐energy X‐ray absorptiometry; L, lumbar spine.

The pQCT results (Table 3) indicated a significant main effect of time for trabecular vBMD (mg/cm^3^) at femur site 4% (CON–BDC4: 329.3 ± 8.1 mg/cm^3^; R1: 326.4 ± 8.4 mg/cm^3^; EX–BDC4: 342.3 ± 7.8 mg/cm^3^; R1: 337.9 ± 8.0 mg/cm^3^; *P* = 0.05) and for cortical vBMD (mg/cm^3^) at femur site 25% (CON–BDC4: 1102.7 ± 6.7 mg/cm^3^; R1: 1095.7 ± 7.1 mg/cm^3^; EX–BDC4: 1089.5 ± 6.7 mg/cm^3^; R1: 1087.5 ± 7.1 mg/cm^3^; *P* = 0.05). However, these changes (ranging from −0.18% to −1.29%) were within the precision error of pQCT measurements.

**TABLE 3:** Femur compartment trabecular and cortical bone outcomes measured with pQCT scan

	Control		Exercise		*P*		
Variable	BDC4	R1	BDC4	R1	Time	Group	Time × Group
Femur compartment 4%							
Total vBMD (mg/cm^3^)	313.9 ± 11.5	316.9 ± 11.5	344.6 ± 10.9	328.1 ± 11.0	0.17	0.12	0.06
Trabecular vBMD (mg/cm^3^)	329.3 ± 8.1	326.4 ± 8.4	342.3 ± 7.8	337.9 ± 8.0	**0.05**	0.83	0.66
Trabecular area (mm^2^)	2225.4 ± 142.9	2232.2 ± 153.1	2289.8 ± 136.3	2398.1 ± 145.9	0.12	0.55	0.17
Femur compartment 25%							
Total vBMD (mg/cm^3^)	481.7 ± 25.9	477.1 ± 25.7	519.0 ± 25.9	516.8 ± 25.7	0.37	0.21	0.74
Cortical vBMD (mg/cm^3^)	1102.7 ± 6.7	1095.7 ± 7.1	1089.5 ± 6.7	1087.5 ± 7.1	**0.05**	0.38	0.26
Cortical area (mm^2^)	375.5 ± 19.7	375.7 ± 20.2	410.0 ± 19.7	411.3 ± 20.2	0.66	0.43	0.74
Cortical thickness (mm)	3.1 ± 0.1	3.0 ± 0.1	3.2 ± 0.1	3.2 ± 0.1	0.85	0.72	0.80
SSI (mm^3^)	7341.5 ± 699.2	7387.0 ± 720.0	7412.0 ± 699.2	7504.1 ± 720.0	0.17	0.61	0.64

*Note*: Values presented as means ± SD. *P*‐values shown in bold indicate statistical significance. Abbreviations: vBMD, volumetric bone mineral density; SSI, stress–strain index.

## 4 DISCUSSION

The present study examined the impact of 14 days of 6° head‐down tilt bed rest (HDBR) and subsequent recovery on serum biomarkers of bone turnover, DXA‐derived BMD (at the whole‐body, hip, femoral neck, femoral trochanters and lumbar spine) and pQCT‐derived bone structure of the femur in healthy older adults, and tested the effects of a multimodal exercise countermeasure intervention on these skeletal outcomes. Our findings demonstrate that 14 days of unloading via HDBR induced a significant increase in serum markers of bone resorption, while recovery was associated with an increase in serum markers of bone formation and both intact and uncarboxylated osteocalcin. There were no changes in DXA‐derived BMD in response to HDBR or subsequent recovery at any of the sites examined. Finally, there was a small decrease in trabecular vBMD at femur site 4% and cortical vBMD at femur site 25% in response to HDBR; however, these small changes in bone structure were within the precision error of pQCT measurements. There was no effect of the exercise countermeasure on any of the outcomes examined.

Blood‐based biomarkers of bone resorption, namely CTX and NTX, increased significantly following bed rest (at R1), suggesting osteoclast activation in response to skeletal unloading in older adults. These results partially align with previous studies showing that biomarkers of bone resorption are elevated within 48 h of bed rest. For example, Baecker et al. (2003) reported that urinary CTX and NTX concentration significantly increased by day 2 of bed rest in healthy young males (25.5 ± 2.9 years), suggesting an early shift toward bone resorption (Baecker et al., 2003). Similarly, Kim et al. (2003) found significant increases in urinary deoxypyridinoline (Dpyr) and NTX after 14 days of HDBR in 11 healthy young males (22 ± 1 years), suggesting accelerated bone resorption and uncoupling from bone formation (Kim et al., 2003). In contrast, early changes in serum CTX and NTX concentration (e.g., by HDT9) were not observed in the present study. This may be attributed to the slower bone turnover associated with ageing (Schini et al., 2023), in contrast to younger populations (average age ∼24 years) in whom bone remodelling occurs more rapidly (Buehlmeier et al., 2017). In the present study, biomarkers of bone formation exhibited a delayed response. Serum P1NP, a biomarker of collagen synthesis and early osteoblast activity, increased significantly during the recovery phase, with elevations evident at 4 weeks (4W) post‐HDBR and persisting through the 4 months (4M) of follow‐up. This rebound effect may suggest a reactivation of bone formation once mechanical loading was restored. These findings are in line with previous literature reporting suppressed or delayed osteoblastic activity during unloading, with recovery or re‐ambulation necessary to re‐stimulate bone formation pathways (Lueken et al., 1993; Morgan et al., 2012). Serum BSAP concentrations followed a similar pattern, with significant increases observed at both the 4W and 4M time points, further supporting the notion that bone formation is not immediately responsive to short‐term unloading and may require extended recovery to normalize or surpass baseline levels (Lueken et al., 1993; Morgan et al., 2012). Additionally, both intact and uncarboxylated osteocalcin concentrations significantly increased only during the recovery phase, with changes evident at 4W post‐HDBR compared to baseline (BDC4). These findings align with those of Yang et al. (2014), who reported an increase in total osteocalcin in young adult males (25–40 years) 10 days after a 60‐day bed rest period, with or without a resistive vibration exercise countermeasure (Yang et al., 2014).

From a bone imaging perspective, the present study demonstrated no significant changes in BMD as assessed by DXA at the regions examined. Specifically, total body, total hip, femoral neck, femoral trochanters and lumbar spine (L1–L4) BMD values remained stable in response to the bed rest and recovery periods (Table 2). These findings are consistent with previous studies reporting the limited sensitivity of DXA in detecting early skeletal adaptations to unloading. For example, a 60‐day bed rest study in young and middle‐aged adults (aged 32 ± 4 years) reported no significant changes in femoral neck BMD (Smith et al., 2008). Similarly, Deutz et al. (2013) observed no significant changes in total BMD following 10 days of bed rest in older adults, further reinforcing the limited ability of DXA to detect early or short‐term bone loss associated with disuse.

To our knowledge, this is the first study to assess femoral bone structure using pQCT in older adults exposed to short‐term HDBR. pQCT results showed no meaningful changes in femoral bone outcomes following 14 days of HDBR. Although small numerical changes were observed in trabecular vBMD at the 4% femur site and cortical vBMD at the 25% femur site, these differences were within the precision error of pQCT and should therefore not be interpreted as biologically meaningful changes. These findings are also in line with previous short‐term bed rest studies reporting no detectable changes in bone mineral density after short periods of unloading (Swinford & Warden, 2010). Together, these results suggest that 14 days of HDBR may be sufficient to detect an increase in circulating biomarkers of bone resorption, but may be too brief to induce measurable changes in femoral bone density or structure using standard pQCT. Application of high resolution pQCT (HR‐pQCT) may be more sensitive to early unloading‐induced skeletal deterioration than conventional pQCT and detect microarchitectural changes that precede measurable density loss in response to short‐term unloading via bed rest.

The absence of a clear protective effect of exercise should also be interpreted in light of the short intervention duration. Previous longer‐duration bed rest studies have reported more pronounced skeletal changes and greater potential for exercise countermeasures to preserve bone outcomes. For example, Armbrecht et al. (2010) showed that daily resistive vibration exercise partially mitigated increases in bone resorption markers, while Rittweger et al. (2010) reported that 56 days of resistive vibration training reduced cortical bone loss and preserved tibial bone strength in young men. These findings suggest that longer unloading periods may be needed to detect structural bone loss and to determine whether exercise can meaningfully protect bone density or structure (Armbrecht et al., 2010; Rittweger et al., 2010). Overall, the present findings suggest that in healthy older adults, 14 days of HDBR and skeletal unloading increases biomarkers associated with bone resorption, but does not alter DXA‐derived BMD across various sites or pQCT‐derived bone structure at the femur. Longer‐duration studies and/or more sensitive imaging methods (e.g., HR‐pQCT) may be required to determine whether structural bone changes emerge over time and whether multimodal in‐bed exercise can effectively preserve bone health during prolonged unloading.

Several limitations of the present study should be acknowledged. First, the sample size was relatively small (n = 22), which may have limited the statistical power to detect between‐group differences on bone‐related outcomes. Second, the duration of bed rest (14 days) was relatively short compared to protocols used in space medicine, and may therefore not capture the long‐term effects of physical inactivity and immobilization on bone health. This is especially relevant given the slower rate of bone turnover relative to muscle or fat tissue (Bonewald, 2019). Third, although the study included both male and female participants, the sample was composed of relatively healthy and physically active older adults; thus, the findings may not generalize to more frail or comorbid populations. Fourth, while the exercise countermeasure was well‐tolerated and showed some protective effects on other physiological and structural outcomes (Dulac et al., 2025; Hajj‐Boutros et al., 2023), it may not have elicited sufficient mechanical strain to fully counteract disuse‐induced bone resorption (Kramer et al., 2017). Fifth, DXA and pQCT are relatively insensitive for detection of short‐term (e.g., 14 days) changes in bone because they reflect the cumulative balance of bone resorption and formation rather than active remodelling. In older adults, age‐related reductions in turnover and ongoing secondary mineralization may further limit the ability of these imaging techniques to detect measurable changes in bone mineral density or structure over such a short timeframe. Finally, the study was not specifically powered to detect an effect of bed rest or an exercise countermeasure on bone‐related outcomes. As a result, the observed effects should be interpreted with caution.

In conclusion, 14 days of head‐down tilt bed rest in older adults, with or without an exercise countermeasure, resulted in an increase in select circulating biomarkers of bone resorption, while subsequent recovery led to an increase in select circulating biomarkers of bone formation. There were no changes in bone imaging‐based outcomes derived from DXA or pQCT. These findings may have important implications for mitigating bone loss during hospitalization, prolonged inactivity, and spaceflight analogue conditions. Future research with larger cohorts and prespecified primary endpoints focused on bone‐related outcomes should investigate the combined effects of exercise and pharmacological strategies, such as antiresorptive or anabolic therapies, to optimize skeletal protection during periods of mechanical unloading in ageing populations.

## AUTHOR CONTRIBUTIONS

Study conception and design: all authors. Acquisition of data: Guy Hajj‐Boutros, Vita Sonjak, Andréa Faust, Carmelo Mastrandrea and José A. Morais. Analysis and interpretation of the data: Guy Hajj‐Boutros, Vita Sonjak, Andréa Faust, Carmelo Mastrandrea, Tyler A. Churchward‐Venne, Richard Kremer and José A. Morais. Drafting of the manuscript: Guy Hajj‐Boutros, Tyler A. Churchward‐Venne, and José A. Morais. Statistical analysis: Guy Hajj‐Boutros, Stéphanie Chevalier, Tyler A. Churchward‐Venne. Review of the manuscript: all authors. All authors have read and approved the final version of this manuscript and agree to be accountable for all aspects of the work in ensuring that questions related to the accuracy or integrity of any part of the work are appropriately investigated and resolved. All persons designated as authors qualify for authorship, and all those who qualify for authorship are listed.

## CONFLICT OF INTEREST

None declared.

## Data Availability

Any requests for data can be sent to the corresponding author.
